# Impact of Autologous Breast Reconstruction on Bra Fit

**DOI:** 10.21203/rs.3.rs-2891426/v1

**Published:** 2023-05-11

**Authors:** Yen-Tung Liu, Novera H. Khan, Mary Catherine Bordes, Gregory P. Reece, Ashleigh M. Francis, Tzuan A. Chen, Karen Bravo, Mia K. Markey

**Affiliations:** The University of Texas at Austin; The University of Texas at Austin; The University of Texas MD Anderson Cancer Center; The University of Texas MD Anderson Cancer Center; The University of Texas MD Anderson Cancer Center; University of Houston; The University of Texas at Austin

**Keywords:** Quantitative imaging, Body image, Breast reconstruction, Bra fit, Breast cancer, Quality of life

## Abstract

**Purpose:**

To inform bra design by analyzing 3D surface images of breast cancer patients who underwent autologous breast reconstruction.

**Methods:**

We computed bra design measurements on 3D surface images of patients who underwent unilateral and bilateral autologous breast reconstruction. Breast measurements and right-left symmetry between preoperative baseline and postoperative time points were compared using either paired Student *t* test or Wilcoxon signed rank test, depending on the data's distribution. Regression analysis determined associations between measurements and patient characteristics such as age. Postoperative measurements and symmetry differences were also compared between autologous and implant-based breast reconstruction.

**Results:**

Among participants who underwent bilateral autologous breast reconstruction, the reconstructed breasts were smaller and positioned higher on the chest wall than their native breasts. For patients who underwent unilateral reconstruction, similar postoperative changes were observed in the contralateral breast due to symmetry procedures. Overall, for participants whose baseline breast measurements showed substantial asymmetry, unilateral reconstruction decreased right-left asymmetry whereas bilateral reconstruction amplified right-left asymmetry. Preoperative baseline breast measurements, age, and BMI were statistically significantly associated with most postoperative breast measurements for participants who underwent bilateral autologous reconstruction. Compared to implant-based reconstruction, autologous reconstruction resulted in fewer changes in breast shape and symmetry that are pertinent to bra fit.

**Conclusion:**

Preoperative baseline breast measurements, age, and BMI can impact bra designs for breast cancer survivors who undergo autologous reconstruction due to size, shape, and symmetry changes. Bra needs of people who undergo autologous reconstruction differ from those who undergo implant-based reconstruction.

## Introduction

Loss of breasts due to breast cancer and its treatment can negatively affect a person’s quality of life in multiple ways, including their body image [1-5] and sexual well-being [1-4]. By re-creating a breast form that is satisfying to the patient, breast reconstruction can mitigate detrimental changes in quality of life associated with complete or partial mastectomy [1, 6-9]. Many surgical techniques can be employed for breast reconstruction [10-12], with most falling into the broad categories of implant-based or autologous reconstruction. In an implant-based reconstruction, a breast mound is created by inserting an implant filled with saline or silicone gel under the breast skin. In autologous reconstruction, a breast mound is created using tissue obtained from another part of the person’s body, such as the abdomen, thighs, or back.

The clothing one wears has a significant impact on the experience of one’s body [13, 14]. The clothing of most feminine-presenting individuals includes a brassiere (bra) [15]. A bra is a foundational garment that not only covers and supports the breasts but impacts the fit of the clothing worn over them [16-18], and difficulty finding a bra that fits can cause physical and psychological distress [19, 20]. People with atypical breast shape, size, or symmetry often have difficulty finding a bra that is comfortable, supportive, and well-fitting off the shelf [21]. Ill-fitting bras are associated with breast, chest, and shoulder pain; embarrassment; and overall decreased quality of life [21-26]. For breast cancer patients, bra difficulties can impede returning to a sense of normalcy and well-being after cancer [20]. Breast cancer patients experience treatment side effects [27,28] that can influence satisfaction with wearable products, e.g., a lingerie wardrobe that no longer works for their new body. Wearability of a bra and breast sensitivity and asymmetry [29] are important factors for breast cancer survivors’ satisfaction with cosmetic outcomes and body image [30].

In a previous study, we analyzed torso measurements on 3D photographs of patients before and after unilateral and bilateral implant-based breast reconstruction and discussed implications for bra design [31]. In this study, we analyze torso measurements on 3D photographs of patients before and after unilateral and bilateral autologous breast reconstruction and discuss bra design implications for patients who undergo autologous breast reconstruction. We also compare measurement changes and bra design considerations for autologous and implant-based breast reconstruction.

## Material and Methods

### Participants

Participants provided informed consent under institutional review board protocol #2010-0321 approved by The University of Texas MD Anderson Cancer Center. Data included demographics, medical records (e.g., reconstruction details), and 3D surface images (3dMDTorso, 3dMD, Atlanta, GA, USA) of participants’ torsos.

Participants were included in this study if they underwent unilateral or bilateral autologous breast reconstruction and had useable 3D surface images both at a baseline study visit with two native breasts and at least 42 days after the autologous tissue transfer procedure. Our study sample consisted of 11 participants with bilateral autologous breast reconstruction and five participants with unilateral autologous breast reconstruction who met the selection criteria (**Supplementary Table 1**). Details of how we operationalized [32] ethnicity, race, sex, and other demographic characteristics for self-reported data collection and summary statistics are summarized in **Supplementary Table 2**. All participants indicated female as their legal sex. Most participants identified as white and not Hispanic. The mean age of the participants was 53.13 years, and the mean body mass index (BMI) was 29.19. All participants who underwent unilateral autologous reconstruction also had contralateral symmetry procedures performed to match the size and shape of the reconstructed breast.

### Measurements

In a previous study, we identified 8 fiducial points used in bra design that can be localized on clinical 3D surface images of breast reconstruction patients and the corresponding terminology used for those fiducial points in the apparel design and reconstructive surgery communities (Fig, 1) [33].

We (YL and NHK) annotated the eight fiducial points on 3D surface images of participants’ breasts and computed 10 breast measurements based on them. The fiducial point annotations were reviewed by an experienced reconstructive surgeon (GPR). The 10 breast measurements were the straight line distance from front neck point to bust point, the contour distance from under-bust point of breast base to bust point, the contour distance from side point of breast base to bust point, the contour distance from center front point on the bust line to bust point, the contour distance from front point of breast base to bust point, the contour distance from mid-shoulder point to top point of breast base, the contour distance from mid-shoulder point to bust point, the contour distance from top point of breast base to bust point, the straight line distance between the two front points of breast base, and the straight line distance between the two bust points. We also calculated the symmetry for all single-breast measurements between the left and right breasts, i.e., all measurements except the between-breast straight line distances of the front points of breast base and of the bust points. Symmetry was calculated as the shorter measurement divided by the longer measurement such that the symmetry ranges from 0 to 1 with higher values indicating greater symmetry.

### Statistical analysis

Breast measurements were made on the 3D surface images, and the symmetry of those measurements was computed between the right and left breasts. Normality was assessed using the Shapiro-Wilk test and Q-Q plots. Depending on the normality of the data, either the paired Student *t*-test or the Wilcoxon signed rank test was used to compare the breast measurements and the right-left symmetry values for the preoperative baseline vs. postoperative time points. Likewise, the postoperative right-left breast symmetry of patients who underwent unilateral reconstruction was compared to that of patients who underwent bilateral breast reconstruction using either the Student *t*-test or the Wilcoxon signed rank test, depending on the results of normality checks. For comparisons between preoperative baseline and postoperative time points, statistical analyses were performed separately between cohorts of patients who underwent unilateral and bilateral autologous breast reconstruction. In addition, post-operative breast measurements and right-left symmetry were compared between autologous and implant-based breast reconstruction using either the paired Student t-test or the Wilcoxon signed-rank test.

Participants who met the study selection criteria were asked to complete the BREAST-Q questionnaire [34] during a follow-up visit after their reconstruction surgery. Trends in post-operative BREAST-Q measures associated with bra fit were reported for participants who underwent either autologous or implant-based breast reconstruction.

Linear regression analysis was used to investigate the associations between participants’ preoperative characteristics and postoperative breast measurements. Normality was assessed using the Shapiro-Wilk test and Q-Q plots. Generalized estimating equations were used to fit 10 breast measurements individually with the baseline measurement as the independent variable and the corresponding postoperative measurement as the dependent variable. Age, BMI, and days since reconstruction were considered as potential covariates. The significance of each of the independent variables in the model was assessed by the Wald test, and *p*-values < 0.05 were considered statistically significant. Residual plots were used to check the model fit. Variance inflation factor was used to measure multicollinearity between independent variables. Analyses were performed in Python (statsmodels package).

## Results

### Measurements: unilateral reconstruction

**Figure 2:**
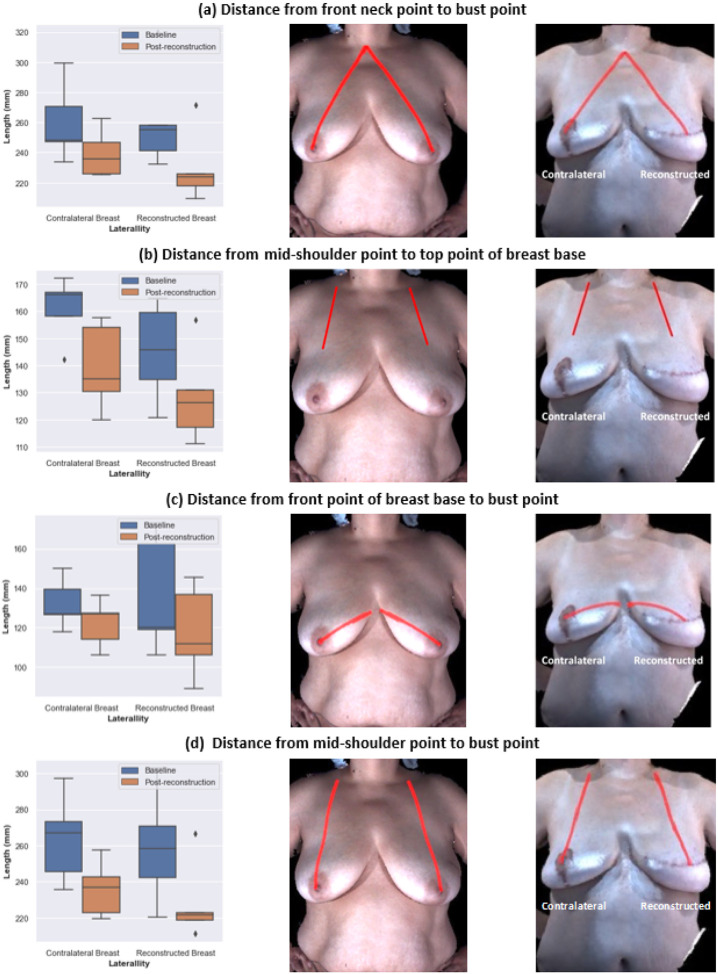
Differences between measurements taken before cancer surgery and after unilateral autologous reconstruction. Left column: Measurements of the contralateral breast and reconstructed breast at baseline and after reconstruction for all participants who underwent unilateral autologous reconstruction. Middle column: Illustration of the measurement on an example patient at baseline. Right column: Illustration of the measurement on an example patient after reconstruction. aThe front neck point to bust point distance statistically significantly decreased in both reconstructed breasts and contralateral breasts after unilateral autologous reconstruction. b The mid-shoulder point to top point of breast base distance statistically significantly decreased in both reconstructed breasts and contralateral breasts after unilateral autologous reconstruction. c The front point of breast base to bust point distance statistically significantly decreased in reconstructed breasts after unilateral autologous reconstruction. d The mid-shoulder point to bust point distance statistically significantly decreased in contralateral breasts after unilateral reconstruction.

The reconstructed breasts of participants who underwent unilateral breast reconstruction were grouped together and compared to their contralateral breasts. A statistically significant decrease in the measurements of the reconstructed breasts relative to the native breasts was observed for the following: the distance from front neck point to bust point, the distance from front point of breast base to bust point, and the distance from mid-shoulder point to top point of breast base. For the contralateral breasts, the distance from front neck point to bust point, the distance from mid-shoulder point to top point of breast base, and the distance from mid-shoulder point to bust point statistically significantly decreased after surgery relative to the native breasts (Fig. 2). The under-bust point of breast base was not visible in four of the five baseline images and three of the five post-reconstruction images, which is typical because ptosis is common for people of the ages in this study. Breast ptosis refers to the descent of the nipple relative to the inferior boundary of the breast (inframammary fold) due to age, weight changes, higher BMI, etc. [35]. The changes in breast measurements indicate that the reconstructed breasts were typically smaller and positioned higher on the chest wall compared to the native breasts. Similar postoperative changes were observed in the contralateral breasts due to symmetry procedures. Note that four of the five participants who underwent unilateral reconstruction had completed contralateral symmetry procedures at least three months prior to the postoperative images assessed in this study. Further details about the analysis of unilateral breast reconstruction measures are shown in **Supplementary Fig. 1** and **Supplementary Tables 3 and 4**.

### Measurements: bilateral reconstruction

**Figure 3:**
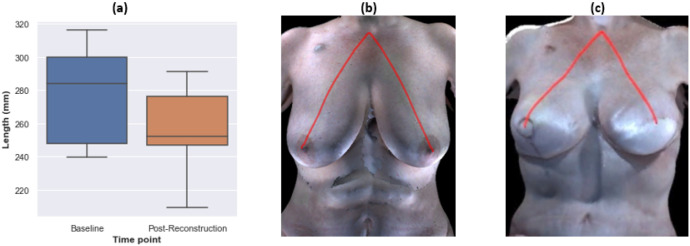
The distance from front neck point to bust point statistically significantly decreased in both right and left breasts after bilateral autologous reconstruction. a Boxplot of the measurements at the baseline and after reconstruction for all participants who underwent bilateral autologous reconstruction. b Illustration of the measurement on an example patient at baseline. c Illustration of the measurement on an example patient after reconstruction.

For the participants who underwent bilateral breast reconstruction, breasts were grouped into right and left as both were reconstructed. The only measurement that was statistically significantly different at the postoperative time point relative to the preoperative baseline was the distance from front neck point to bust point, which decreased after reconstruction (Fig. 3). For transparency, the results for the other measures are provided in **Supplementary Fig. 2**. The results were consistent between the left and right breasts. Thus, similar to our findings for unilateral reconstruction, the breast measurements of participants who underwent bilateral autologous reconstruction indicate that their reconstructed breasts were generally smaller and positioned higher on the chest wall than were their native breasts. However, compared to the unilateral reconstruction cases, fewer measurements were statistically significantly different between the preoperative baseline and postoperative time points for the bilateral autologous reconstruction cases. The under-bust point of the breast base was not visible in three of the 11 baseline images and three of the 11 post-reconstruction images due to ptosis. **Supplementary Tables 5 and 6** present the summary statistics and results of hypothesis testing for the right and left breasts, respectively.

### Symmetry

**Figure 4:**
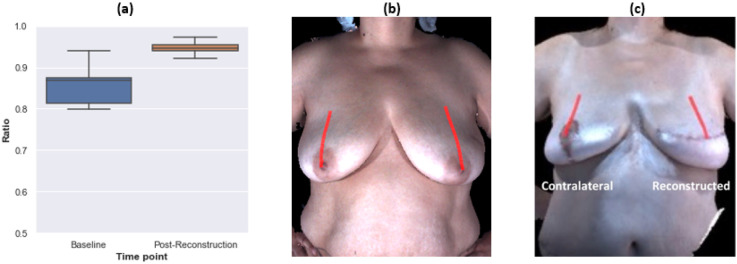
Symmetry of the distance from top point of breast base to bust point statistically significantly increased after unilateral autologous reconstruction. a Boxplot of the symmetry ratio at baseline and after reconstruction for all unilateral autologous participants. b Illustration of the measurement on an example patient at the baseline. c Illustration of the measurement on an example patient after reconstruction.Note: Symmetry is defined such that it ranges from 0 to 1, with higher values indicating more symmetry. Since the patient population in this study showed high levels of symmetry, we present the symmetry over the range from 0.5 to 1 for visual clarity.

**Figure 5:**
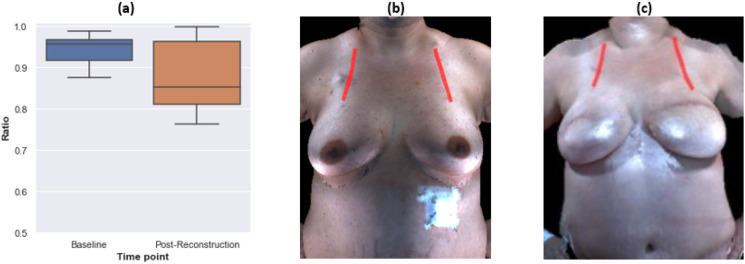
Symmetry of the distance from mid-shoulder point to top point of breast base statistically significantly decreased after bilateral autologous reconstruction. a Boxplot of the symmetry ratio at baseline and after reconstruction for all participants who underwent bilateral autologous reconstruction. b Illustration of the measurement on an example patient at baseline. c Illustration of the measurement on an example patient after reconstruction. Note: Symmetry is defined such that it ranges from 0 to 1, with higher values indicating more symmetry. Since the patient population in this study showed high levels of symmetry, we present the symmetry over the range from 0.5 to 1 for visual clarity.

Symmetry between the right and left breasts was comparable for both unilateral and bilateral autologous reconstructions relative to the symmetry between the participants’ preoperative native breasts. For the participants who underwent unilateral breast reconstruction, there were no statistically significant differences between most pre- and post-operation symmetry measurements (**Supplementary Table 7**). However, the symmetry of the distance from the top point of breast base to bust point increased statistically significantly after reconstruction due to contralateral symmetry procedures and reduced ptosis (Fig. 4). For the participants who underwent bilateral breast reconstruction, there were no significant differences between most pre- and post-operation symmetry measurements (**Supplementary Table 8**). However, symmetry of the distance from the mid-shoulder point to top point of breast base decreased statistically significantly after reconstruction (Fig. 5). No statistically significant difference in symmetry outcomes was observed in the comparisons of unilateral and bilateral reconstruction cases (**Supplementary Table 9**). Overall, among the participants whose baseline breast measurements showed substantial asymmetry, unilateral reconstruction decreased right-left asymmetry. In contrast, bilateral reconstruction amplified right-left asymmetry in this data set.

### Regression analysis

Preoperative baseline breast measurements, age, and BMI were statistically significantly associated with most postoperative breast measurements for the participants who underwent bilateral autologous reconstruction (**Supplementary Table 10**). Older age was significantly associated with shorter postoperative distance from the front point of breast base to bust point, as well as greater postoperative distance between front points of breast bases, which appeared to delineate breast orientation from the midline. There was a significant correlation between a greater BMI and greater postoperative distance in following measurements: from front neck point to bust point, distance from side point of breast base to bust point, distance from center front point on the bust line to bust point, distance from front point of breast base to bust point, distance from mid-shoulder point to top point of breast base, distance from mid-shoulder point to bust point, and the distance between bust points. Taken together, these findings indicate that BMI is associated with broader changes breast shape as well as to breast size. Preoperative baseline breast measurements were statistically significantly associated with postoperative measurements for the distance from front neck point to bust point, distance from center front point on the bust line to bust point, distance from front point of breast base to bust point, distance from mid-shoulder point to top point of breast base, and the distance between bust points. Thus, the preoperative baseline measurements were associated with where the postoperative breast is located on the chest wall and the postoperative breast shape. The number of days since reconstruction was not associated with postoperative breast measurements, which makes sense because breast shape and size change little once patients are no longer in active care. Due to the small sample size (n=5), regression analysis was not performed on participants who underwent unilateral breast reconstruction.

## Discussion

### Implications for bra design: bra strap design

The distance from the mid-shoulder point to the top point of the breast base significantly decreased postoperatively, indicating that reconstructed breasts formed by autologous procedures are smaller and positioned higher on the chest wall than native breasts (Fig. 2b). Likewise, we observed a statistically significant decrease in the distance from the front neck point to the bust point (Fig. 2a and Fig. 3). Previously, we reported a similar outcome for participants who underwent implant-based breast reconstruction [36].

The distance from the mid-shoulder point to the bust point significantly decreased in the contralateral breasts since patients undergoing unilateral autologous reconstruction also underwent symmetry procedures on the contralateral breast (Fig. 2d). Hence, the right-left symmetry for participants who underwent autologous breast reconstruction was about the same after reconstruction as it was for their native breasts, with the exception of the distance from the mid-shoulder point to the top point of breast base (**Supplementary Table 8**). For participants who underwent bilateral autologous breast reconstruction, the mean symmetry for the distance from the mid-shoulder point to the top point of breast base statistically significantly decreased, from 0.94 to 0.87 (median symmetry decreased from 0.96 to 0.85), indicating that one of the breasts is typically positioned higher on the chest wall than the other breast is after autologous reconstruction (Fig. 5). These observations reflect the difficulty of predicting how far a reconstructed breast will descend on the chest during the first few postoperative months. Moreover, two breasts on the same patient often descend to different heights and at different rates. Hence, it is more challenging to mitigate substantial preoperative asymmetry through the reconstruction procedure when the surgeon must position two reconstructed breasts rather than one reconstructed breast.

Differences in the vertical positions of the breasts after autologous reconstruction can be somewhat accommodated for in bra fitting because the length of the bra straps is adjustable. Further accommodation of breast changes resulting from breast cancer treatment with autologous reconstruction could be achieved by enabling customization of the width of bra straps. Customization of bra strap width would help balance the strap/shoulder interface pressure between the left and right sides of the body.

### Implications for bra design: bra cup shape

Our previous study [36] of participants who underwent implant-based breast reconstruction reported postoperative changes in four breast measurements relative to the native breasts: the top point of breast base to the bust point, the front point of the breast base to the bust point, the under-bust point of the breast base to the bust point, and the side point of the breast base to the bust point. We argued that changes to the bra cup shape may be needed to accommodate the breast shape changes associated with implant-reconstruction. In contrast, in the present analysis of participants who underwent autologous breast reconstruction, we observed that only one measurement, the distance from the front point of the breast base to the bust point, statistically significantly decreased after surgery (Fig. 2c). Overall, participants in our study experienced less change in breast shape with autologous reconstruction than previously reported for implant-based reconstruction [36], which makes sense because autologous reconstruction is performed using the patient’s own tissue whereas in implant-based reconstruction the shape of the implant itself remains apparent despite overlying tissues. Consequently, compared with breast cancer survivors who undergo implant-based reconstruction, survivors who undergo autologous reconstruction may more readily find a bra whose cup shape fits their postoperative breasts.

### Implications for bra design: right-left symmetry

Compared to our prior study of participants who underwent unilateral implant-based breast reconstruction [36], participants who underwent unilateral autologous breast reconstruction showed statistically significantly better symmetry outcomes with respect to the distance from the side point of the breast base to the bust point and the distance from the top point of the breast base to the bust point (**Supplementary Table 11**). Patients may even experience increased right-left symmetry after a unilateral autologous reconstruction compared to the right-left symmetry of their native breasts at baseline due to contralateral symmetry procedures, particularly if there was marked right-left asymmetry of their native breasts at baseline (Fig. 4, **Supplementary Table 7**).

Our comparison between the symmetry outcomes of participants who underwent bilateral implant-based breast reconstruction [36] and those who underwent bilateral autologous reconstruction was less conclusive. Bilateral implant-based breast reconstruction showed statistically significantly more symmetry than did bilateral autologous breast reconstruction cases with respect to the distance from the side point of the breast base to the bust point, but a similar trend was not evident for the other measures (**Supplementary Table 12**).

Our prior study of implant-based reconstruction cases [36] demonstrated statistically significantly higher symmetry outcomes in bilateral reconstruction relative to unilateral reconstruction in all breast measurements except for the distance from the front point of the breast base to the bust point and the distance from the under-bust point of the breast base to the bust point (**Supplementary Table 13**). In contrast, laterality did not statistically significantly influence breast measurement symmetry for participants who underwent autologous breast reconstruction in the present study (**Supplementary Table 9**).

Taken together, these observations about right-left symmetry suggest that when unilateral reconstruction is to be performed, autologous reconstruction may offer more postoperative symmetry than implant-based reconstruction. This finding is consistent with our previous study [37] using surgical measurements that do not directly correlate with apparel design measures. Hence, since off-the-rack bras are designed assuming a high degree of right-left symmetry, breast cancer survivors who undergo a unilateral autologous breast reconstruction may have less difficulty in finding a bra that fits than will those who undergo a unilateral implant-based breast reconstruction. When a bilateral reconstruction is performed, there is not a clear symmetry advantage between implant-based and autologous reconstructions that would influence bra fit, though implant-based reconstructions still pose challenges with respect to bra cup shape as summarized above and discussed in detail in our prior study [31].

#### Quality of Life

Compared to our sample of patients who underwent implant-based breast reconstruction, participants in our sample who underwent autologous breast reconstruction showed a trend toward better satisfaction with outcomes, and greater satisfaction with their breasts, as measured by the BREAST-Q [34], [31] (**Supplementary Fig. 3**). This observation is aligned with the literature on breast reconstruction psychosocial outcomes [1, 6], which consistently reports better long-term quality of life outcomes for patients who undergo autologous breast reconstruction compared with those who undergo implant-based breast reconstruction. Given typical postoperative changes in breast measurements and their impact on bra fit, future research should explore more carefully the relationship between bra experiences and quality of life outcomes in patients undergoing different types of breast reconstruction. As a starting point, BREAST-Q [34] includes measures of comfort while wearing bras; overall satisfaction with the breast area, clothed and unclothed; and the ability to wear fitted clothing. For example, compared to our previous sample of patients who underwent implant-based breast reconstruction [31], participants in our sample who underwent autologous breast reconstruction showed a trend toward endorsing more bra comfort, as measured by the BREAST-Q (**Supplementary Fig. 4**). In addition, future work should investigate the potential of new bra designs for mitigating differences in quality of life experienced by patients who undergo different types of reconstruction.

## Conclusion

Bra designs for breast cancer survivors who undergo autologous reconstruction procedures should account for typical changes in the size, shape, and location of the breast mounds. Bra needs of people who undergo autologous reconstruction differ from those who undergo implant-based reconstruction. Compared to the breast changes that are typical for people who undergo implant-based reconstruction, autologous reconstruction results in fewer breast changes pertinent to bra design. Regression analysis results suggest that breast cancer patients’ preoperative characteristics, such as breast measurements, age, and BMI, are associated with postoperative breast changes for patients who undergo autologous breast reconstruction and hence may impact bra needs after autologous reconstruction. Future investigations of the quality of life outcomes of breast reconstruction should consider bra fit challenges arising from postoperative changes in breast size, shape, and location.

## Figures and Tables

**Figure 1:**
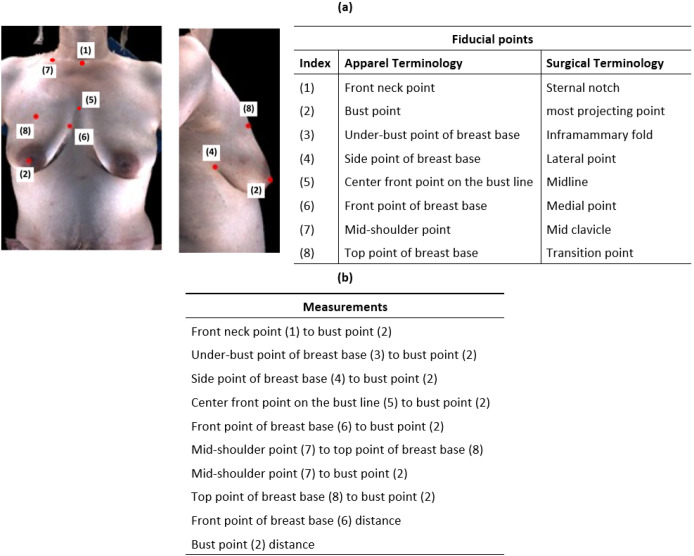
Visual definition of the fiducial points used in this study (a) and measurements calculated based on these points (b). Correspondence between the fiducial points in the apparel design and reconstructive surgery disciplines [33] (a). As is the case in this illustration, the under-bust point of breast base is often not visible in photographs of women with ptosis.
